# Intranasal Administration of Recombinant TRAIL Down-Regulates CXCL-1/KC in an Ovalbumin-Induced Airway Inflammation Murine Model

**DOI:** 10.1371/journal.pone.0115387

**Published:** 2014-12-15

**Authors:** Veronica Tisato, Chiara Garrovo, Stefania Biffi, Francesca Petrera, Rebecca Voltan, Fabio Casciano, Germana Meroni, Chiara Agnoletto, Giorgio Zauli, Paola Secchiero

**Affiliations:** 1 Department of Morphology, Surgery, Experimental Medicine and LTTA Centre, University of Ferrara, Ferrara, Italy; 2 Institute for Maternal and Child Health, IRCCS “Burlo Garofolo”, Trieste, Italy; 3 Cluster in Biomedicine, CBM S.c.r.l., Area Science Park, Trieste, Italy; French National Centre for Scientific Research, France

## Abstract

Ovalbumin (OVA)-sensitized BALB/c mice were i.n. instilled with recombinant TNF-related apoptosis inducing ligand (TRAIL) 24 hours before OVA challenge. The total number of leukocytes and the levels of the chemokine CXCL-1/KC significantly increased in the bronchoalveolar lavage (BAL) fluids of allergic animals with respect to control littermates, but not in the BAL of mice i.n. pretreated with recombinant TRAIL before OVA challenge. In particular, TRAIL pretreatment significantly reduced the BAL percentage of both eosinophils and neutrophils. On the other hand, when TRAIL was administrated simultaneously to OVA challenge its effect on BAL infiltration was attenuated. Overall, the results show that the i.n. pretreatment with TRAIL down-modulated allergic airway inflammation.

## Introduction

Asthma is a common syndrome with increasing prevalence in both children and adults [1]. Despite the increasing prevalence and socioeconomic burden, the underlying pathophysiology remains poorly defined in a large percentage of asthmatics. Asthma is indeed a complex syndrome with many clinical phenotypes, usually characterized by chronic airway inflammation associated with airway obstruction and hyperresponsiveness (AHR) [1]. Airway inflammation is thought to play an important role in mediating airflow obstruction and the inflammatory changes associated with asthma are usually characterized by an accumulation of eosinophils, T lymphocytes, mast cells and neutrophils in the airway walls, lung tissue and airway lumen [1]. TNF-related apoptosis inducing ligand (TRAIL) is an important immunomodulatory cytokine and a series of studies have addressed the potential role of the TRAIL/TRAIL-receptor system in the context of asthma, mainly in animal models [2]–[9]. While some studies, mainly based on the use of TRAIL^-/-^ knock out mouse models [4], [6], point on a pro-inflammatory role of endogenous TRAIL, a more recent study has demonstrated that recombinant TRAIL administration might rather plays a role in the resolution phase of asthma, by promoting apoptosis of infiltrating leukocytes [9]. These conflicting data are consistent with *in vitro* and *in vivo* studies demonstrating a dual role for TRAIL, which can either function as a pro- or anti-inflammatory cytokine targeting inflammatory cells, participating in either the initiation or in the resolution of inflammatory/immune responses, depending on the context [5], [10], [11].

Based on our experience in characterizing the role of the TRAIL/TRAIL-receptor system in different physio-pathological contexts [11]–[15], in the present study we have evaluated the effect(s) of intranasal treatment with recombinant TRAIL in a mild allergen-induced airway inflammation model represented by ovalbumin (OVA)-sensitized BALB/c mice [16]–[18].

## Material and Methods

### Experimental allergen-induced airway inflammation model

Pathogen-free female BALB/c mice (4- to 6-weeks old) were purchased from Charles River Laboratories Inc. (Milano, Italy). Mice were housed in a controlled environment and maintained with ad libitum food and water. All animal procedures were used to minimize animal pain and suffering. All the experimental procedures were performed in strict accordance with the recommendations in the Guide for the Care and Use of the Laboratory Animals of the National Institutes of Health and in compliance with the European (86/609/EEC) and Italian (D.L.116/92) laws. The protocols for mice experimentation were approved by the Institutional Animal Care and Use Committee of the Cluster in Biomedicine (CBM) of the Area Science Park of Trieste and by the Italian Ministry of Health (DM 17/2001-A dd. 02/02/2011).

For the experimental allergen-induced airway inflammation model, BALB/c mice were sensitized and challenged by using OVA (Sigma-Aldrich, St Louis, MO) as allergen as previously described [18]. In particular, mice were treated on day 0 and day 21 with 50 µg of OVA in a volume of 0.2 ml PBS (Sigma-Aldrich) *via* intraperitoneal (i.p.) injection. On day 28 and day 29 post-immunization, mice were challenged with intranasal (i.n.) instillation of 250 µg OVA diluted in 25 µl of PBS. All i.n. procedures were performed under mild 2% isoflurane, 2 L/min oxygen anesthesia of the mice. Healthy age and gender matched BALB/c mice were used as healthy controls and underwent to the same schedule of injection with control vehicle (PBS). At different days, blood samples were collected from healthy controls and asthmatic mice for IgE quantification and at day 32 mice were sacrified with an overdose of isoflurane. Blood was collected and lungs were fixed in 10% phosphate-buffered formalin for 24 hours followed by immersion in 70% ethanol, embedded in paraffin. Three µm thick lung sections containing main stem bronchi were stained with hematoxylin and eosin, as previously reported [18], before analyses for the presence of bronchial architecture remodeling and inflammation. To semi-quantitatively evaluate the bronchial architecture, at least 40 airways were scored for each sample and assigned regular versus distorted (irregular arrangement and thickening of the epithelial layer) lining epithelia. The ratio between distorted/regular epithelia was then calculated for each sample. In parallel, semi-quantitative evaluation of inflammation was carried out by assigning the following arbitrary scores: 0, no inflammatory infiltrates; 0.5, inflammatory infiltrates present in up to 1/3 of lung parenchima; 1, inflammatory infiltrates present in more than 1/3 of lung parenchima.

### TRAIL imaging and treatment

Purified human recombinant TRAIL was prepared and tested for the absence of endotoxin by using the ToxinSensor chromogenic LAL endotoxin assay kit (GenScript, Piscataway, NJ), as previously described [19], [20]. For *in vivo*/*ex vivo* imaging experiments, recombinant TRAIL was labeled with N-hydroxysuccinimmide ester of the cyanine 5.5 (Amersham Pharmacia Biotech) to form Cy5.5-TRAIL [21]. Mice were anesthetized with isoflurane at 1.8–2.0 volume % (Sigma), shaved in the abdomen to avoid laser scattering caused by fur, placed in the small-animal time-domain imager Optix MX2 preclinical NIRF-imager (Advanced Research Technologies, Montreal, CA) and maintained under vaporized isoflurane for the entire imaging session. Two-dimensional regions of interest were selected, and laser power, integration time (repetition time of the excitation per raster point) and scan step size were optimized according to the emitted signal. A blank image was acquired before i.n. administration of Cy5.5-TRAIL to record background signal intensity. After i.n. Cy5.5-TRAIL-instillation (20 µg in 25 µl PBS) the animals were monitored at multiple time points. In all imaging experiments, a 670 nm pulsed laser diode with a repetition frequency of 80 MHz and a time resolution of 12 ps light pulse was used for excitation. Fluorescence emission was collected at 700 nm. For *ex vivo* imaging tissue analysis, the last *in vivo* whole-body imaging session was followed by euthanasia of animals and the explanted lungs were collected, washed in PBS (Sigma-Aldrich) and analyzed with the same Optix system. Optical imaging results were analyzed by reporting fluorescence intensity values in normalized counts (NC) representing the photon count for unit excitation laser power and unit exposure time, allowing comparison among different images. For recombinant TRAIL treatments, mice received i.n. instillation of 20 µg of recombinant TRAIL resuspended in 50 µl PBS either before or at the time of OVA challenges.

### Bronchoalveolar lavage (BAL) fluid analyses

BAL collection was performed at the time of sacrifice by injection of 1 ml of PBS through a tracheal cannula inserted into the lung. The recovered solution was then centrifuged for 10 minutes at 400×g at 4°C, cell-free supernatants were collected and stored at −80°C in single-use aliquots for cytokines/chemokines evaluation while the cell pellets were resuspended in 1 ml of PBS and used for cell counts, cytospin preparation/visualization by May-Giemsa staining and multiparametric flow-cytometry. Cell count and viability was determined by Trypan blue dye exclusion, as previously described [22], while the degree of apoptosis was quantified by Annexin V-FITC/propidium iodide (PI) double staining (Immunotech, Marseille, France) followed by flow cytometry analysis, as previously detailed [23], [24].

For multiparametric FACS analyses, an aliquot of the single-cell suspensions obtained from BAL was resuspended in PBS, 0.5% fetal bovine serum (FBS; Gibco Life Technologies, Grand Island, NY) according to standard protocols and used for cell populations' characterization after staining with the following fluorochrome-conjugated monoclonal antibodies: anti-muCD3*ε* (Clone 145-2C11), anti-muCD19 (Clone 1D3), anti-muMHCII (clone 2G9), anti-muGr1 (Clone RB6-8C5) and anti-muCD11c (Clone HL3) (all from BD Pharmingen, San Jose, CA) and anti-muCCR3 (Clone 2.4G2, R&D Systems, Minneapolis, MN). The purified anti-CD16/CD32 antibody blocking reagent (Clone 2.4G2, BD Pharmingen) was also added to the cells for blocking of Fc receptor. Cells were incubated for 20 minutes at 4°C, washed in PBS and fixed in PBS-1% formaldehyde (Sigma-Aldrich) and 0.5% FBS. Data were acquired in a FACS ARIA II (BD Bioscience), analyzed with FlowJo software (TreeStar, Ashland, OR).

### Biochemical measures

Total IgE were measured in murine serum samples by using the Mouse IgE Single Plex Magnetic Bead Kit (Merck Millipore, Billerica, MA). Levels of recombinant human TRAIL and soluble murine TRAIL were measured in serum samples by commercially available ELISA kits specific for human TRAIL (R&D Systems) and murine TRAIL (Cloud-Clone Corp., Houston, TX), respectively in accordance with the manufacturer's instructions. Samples were analyzed in duplicate with an ELISA reader at 450 nm, as previously described [25].

Levels of murine cytokines/chemokines were measured in BAL fluids using the Multiplex MILLIPLEX MAP Mouse Cytokine/Chemokine Magnetic Bead Panel Kit (Merck Millipore) that allowed the simultaneous quantification of the following 32 murine cytokines/chemokines: Eotaxin, G-CSF, GM-CSF, IFN-γ, IL-1α, IL-1β, IL-2, IL-3, IL-4, IL-5, IL-6, IL-7, IL-9, IL-10, IL-12 (p40), IL-12 (p70), IL-13, IL-15, IL-17, IP-10, KC, LIF, LIX, MCP-1, M-CSF, MIG, MIP-1α, MIP-1ß, MIP-2, RANTES, TNF-α, VEGF. All samples were run in duplicate in accordance with the manufacturer's instructions.

### Quantitative RT-PCR

Total RNA was extracted from murine lung tissues using the Qiagen RNeasy Plus mini kit (Qiagen, Hilden, Germany) according to the supplier's instructions, and further treated with the Qiagen RNase-Free DNase Set (Qiagen) to remove genomic DNA. For the integrity measurement, 4 µl of the total RNA were analyzed on an Agilent Cary 60 UV-VisSpectrophotometer (Agilent Technologies Inc., Santa Clara, CA). Total RNA was transcribed into cDNA and amplified using the Express One-Step Superscript qRT-PCR Kit (Invitrogen, Carlsbad, CA). Analysis of murine CXCL1 (KC), CCL20 and Mideline-1 (MID-1) was carried out with validated TaqMan Gene Expression Assays specific PCR primers sets (Invitrogen) [26]. All samples were run in triplicate using the real time thermal analyzer Rotor-Gene 6000 (Corbett, Cambridge, UK). Expression values were normalized to the housekeeping murine gene POLR2A amplified in the same sample.

### Statistical analysis

Descriptive statistics were calculated. For each set of experiments, values were reported as means ± standard deviation (SD) and box plots were used to show the median, minimum and maximum values, and 25th to 75th percentiles. The results were analyzed by using the Mann-Whitney rank-sum test and statistical significance was defined as p<0.05. All statistical analyses were performed with SPSS Statistic 20 software (SPSS Inc., Chicago, IL).

## Results

### *In vivo* biodistribution of recombinant TRAIL upon intranasal instillation

**Figure 1:**
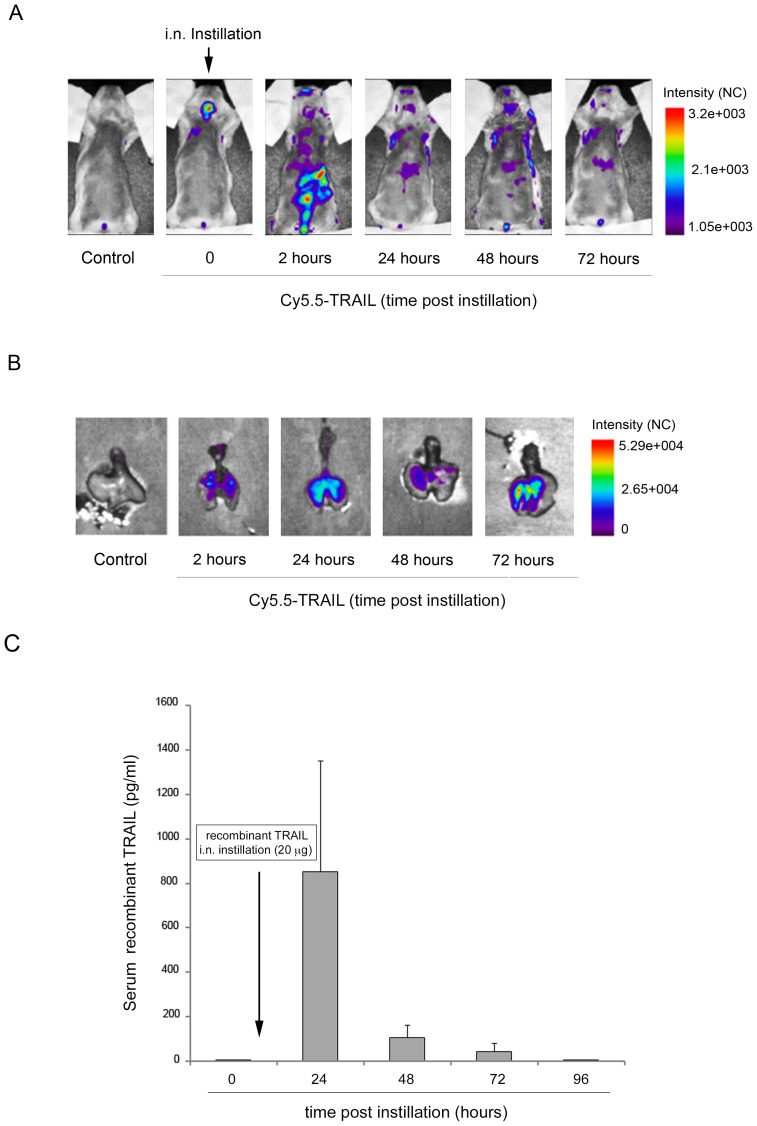
*In vivo* biodistribution of recombinant TRAIL. Mice were i.n. instilled with recombinant TRAIL (20 µg). In **A**, the distribution of the labeled Cy5.5-TRAIL was recorded at the indicated time intervals by whole body scans in six mice. Results of one representative mouse before (control) and after i.n. instillation are shown. In **B**, *ex vivo* representative images of lungs isolated from mice sacrificed at the indicated time points after i.n. instillation of Cy5.5-TRAIL compared to a representative lung isolated from a control mouse (i.n. instillation with control vehicle). In **C**, after i.n. instillation a time course detection of circulating recombinant human TRAIL was assessed by ELISA performed on murine sera samples. Results are reported as mean ± SD of 6 mice.

The primary aim of this study was to explore the potential of TRAIL as pharmacological approach aiming to modulate the inflammatory process associated to allergen-induced airway inflammation. To this end, in a first set of experiments we evaluated the biodistribution of recombinant TRAIL after i.n. delivery by using an *in vivo*/*ex vivo* imaging approach. A single i.n. instillation of Cy5.5-labelled TRAIL (20 µg/instillation) was administered to BALB-c mice and whole-body imaging was performed acquiring fluorescence intensity images up to 72 hours post-instillation. As shown in **Fig. 1A**, soon after i.n. instillation, the *in vivo* analysis displayed the highest fluorescence signal in the upper respiratory tract, while after 2 hours the highest signal resulted associated with gastrointestinal tract and tended to decrease over time. The *ex vivo* analysis of the explanted lungs, performed at different times (2, 24, 48 and 72 hours) after TRAIL-Cy5.5 i.n. delivery, showed a significant and persistent fluorescent signal in the lung that was still significantly detectable 72 hours after i.n. instillation as compared to controls, which showed barely detectable fluorescence (**Fig. 1B**).

Moreover, in order to determine the presence of recombinant TRAIL also at systemic level, we performed a time course analysis after the i.n. delivery, by quantifying recombinant human TRAIL in serum samples using a specific ELISA for human TRAIL detection. As shown in **Fig. 1C**, recombinant human TRAIL was undetectable (below the detection limit: 15.6 pg/ml) at baseline (time 0), became detectable in serum samples at 24 hours (mean ± SD: 852.5±581.7 pg/ml) after i.n. instillation and progressively decreased thereafter (48 hours, mean ± SD: 105.9±57.7 pg/ml; 72 hours, mean ± SD: 43.2±36.1 pg/ml) becoming undetectable at 96 hours. In parallel, the mean baseline levels of soluble murine TRAIL, assessed at all time point examined by using a murine-specific TRAIL ELISA assay, were 727±234 pg/ml.

### Pretreatment with recombinant TRAIL attenuates allergen-induced airway inflammation

**Figure 2:**
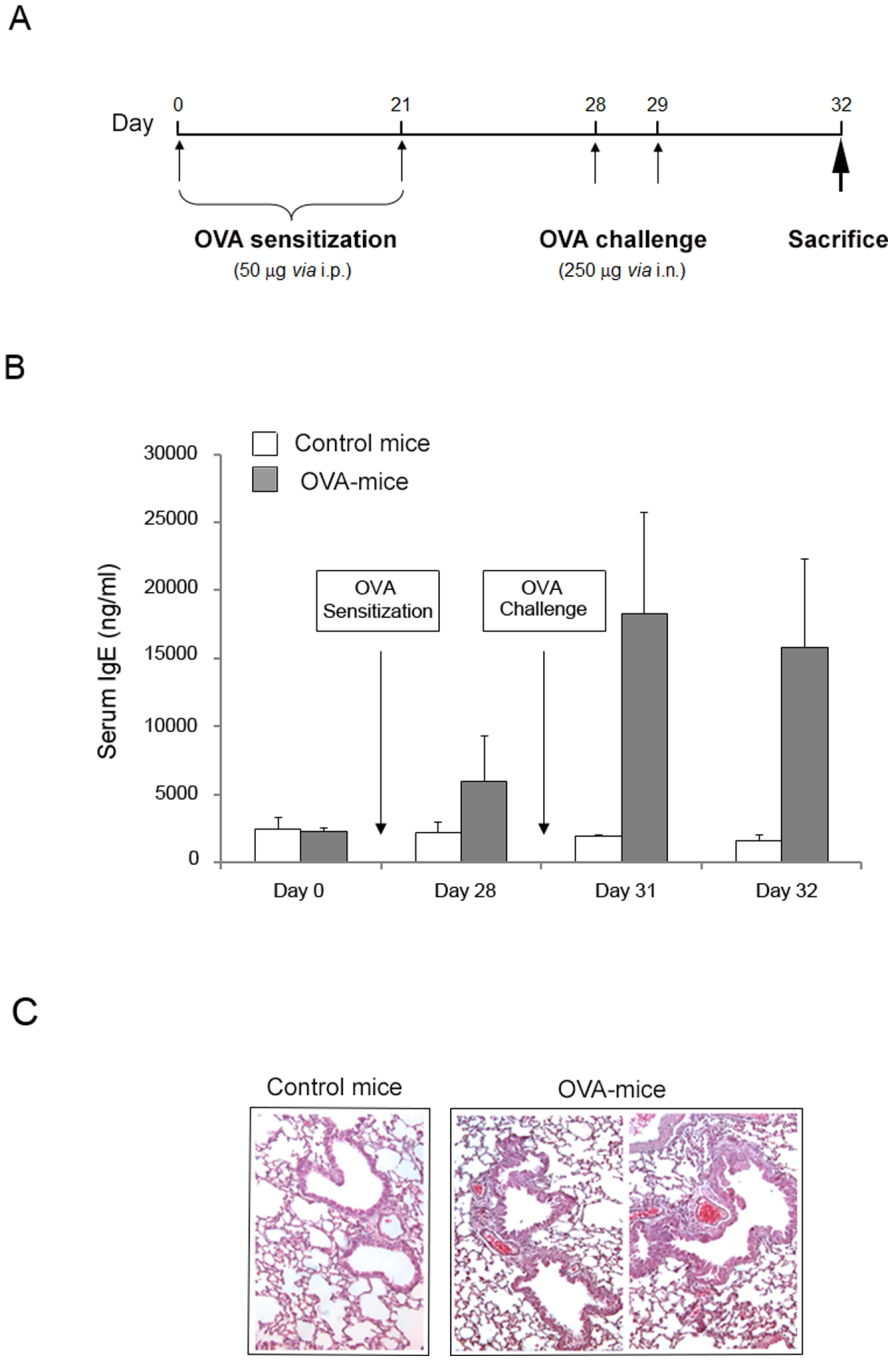
Establishment of airway inflammation mouse model. Mice were exposed to an OVA “sensitization plus challenge” protocol. In **A**, the schedule and the timeline of OVA treatments of BALB/c mice are shown. In **B**, the circulating levels of total IgE were determined in serum samples of controls (n = 16) and OVA-treated mice (n = 16), harvested at the indicated experimental time points. Results are expressed as mean ± SD. In **C**, representative hematoxylin and eosin stained sections of lung tissue isolated (at day 32) from either healthy control or OVA-treated mice. Lungs of OVA-treated mice exhibit airway remodeling and thickness of epithelial cells (original magnification 40X).

OVA-mediated airway inflammation was induced in mice with two rounds of OVA sensitization (50 µg administered by i.p. injection on days 0 and 21) followed by two rounds of OVA challenge (250 µg administered by i.n. instillation on days 28 and 29) (**Fig. 2A**). The development of allergen-induced inflammation in OVA-treated mice was mainly documented by evaluating the presence/levels of circulating IgE in comparison to control mice. Indeed, the exposure of sensitized mice to inhaled antigen elicits a significant (p<0.01) elevation of serum IgE after OVA challenge (**Fig. 2B**) coupled to thickness of airway epithelial cells (**Fig. 2C**).

**Figure 3:**
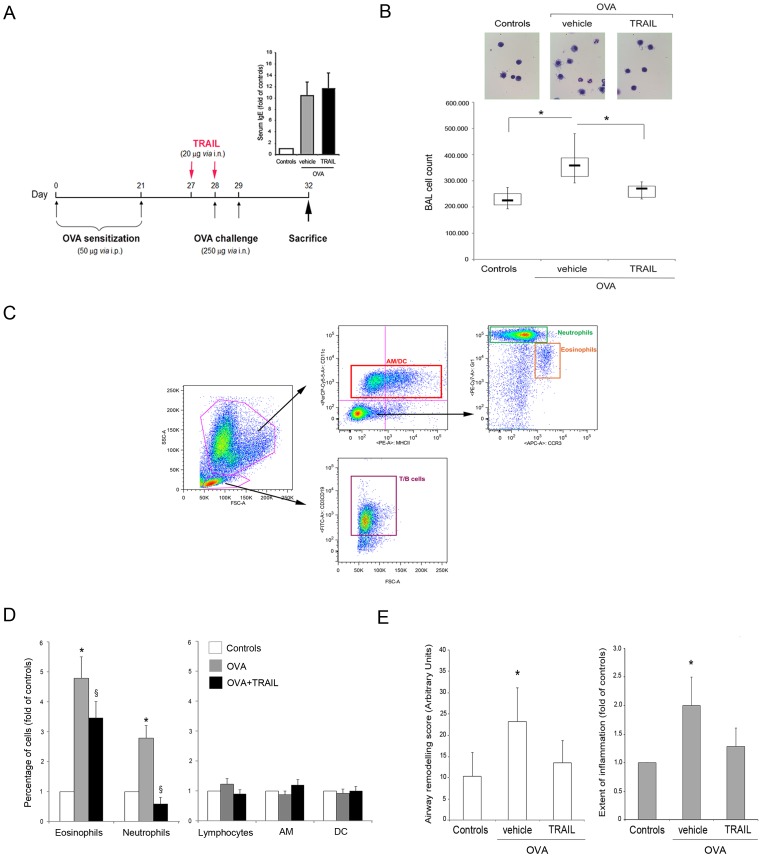
Effect of recombinant TRAIL on OVA-induced airway inflammation. Mice were treated with recombinant TRAIL before OVA challenges and the cellular influx in the BAL was characterized. In **A**, the schedule and the timeline of recombinant TRAIL treatments with regard to OVA sensitizations/challenges of BALB/c mice are shown. At the time of sacrifice (day 32), the circulating levels of total IgE were determined in serum samples of controls, OVA- and TRAIL+OVA-mice and expressed as fold of modulation with respect to controls (**insert**). In **B**, total cell count of cells present in BAL fluids of controls, OVA- and TRAIL+OVA-mice (n = 16 mice/group) is shown. Horizontal bars are median, upper and lower edges of box are 75th and 25th percentiles, lines extending from box are 10th and 90th percentiles; *, P<0.01. Representative images of May-Grünwald Giemsa stained cytospins of BAL cells from each experimental group are shown (original magnification, 20X). In **C**, gating strategy utilized in multiparametric flow cytometry analysis to identify the major BAL infiltrating cell types. In **D**, the percentage of eosinophils, neutrophils, lymphocytes, alveolar macrophages (AM) and dendritic cells (DC) in the BAL of OVA-mice and of TRAIL+OVA-mice is expressed as fold of modulation with respect to the percentages of control healthy mice (set at 1). Results are mean ± SD. *, P<0.05 compared to healthy control mice; ^§^, P<0.05 compared to OVA-mice (Mann-Whitney rank-sum test). In **E**, blinded semi-quantitative analysis of histology from Controls, OVA- and TRAIL+OVA-mice was done to evaluate airway remodeling (**left panel**) and inflammation (**right panel**), scored as detailed in the Material and Methods section. Results are derived from at least 3 microscopy fields for each mouse (n = 8 for each experimental group). Data are expressed as mean ± SD. *, P<0.05 compared to healthy control mice (Mann-Whitney rank-sum test).

In order to evaluate the potential ability of TRAIL in modulating allergen-induced airway inflammation, mice (n = 16/group) were either treated with control vehicle (PBS) or with recombinant TRAIL (20 µg *via* i.n.) on day 27 and 28, before OVA challenges (**Fig. 3A**). In parallel, as controls, healthy mice (n = 6) were instilled with recombinant TRAIL, with the same treatment schedule and protocol. Consistently with the evidence that total cell count in BAL is an indicator of the inflammatory cell influx into the lung from the surrounding blood network, we observed an increase in the absolute number of cells recovered from BAL of OVA-mice at 24 hours post-challenge (median and mean ± SD: 306×10^3^ and 294×10^3^±50×10^3^ cells) with a further increased at 72 hours (median and mean ± SD: 358×10^3^ and 375×10^3^±83×10^3^ cells), reaching significant (p<0.01) differences with respect to the healthy control mice (median and mean ± SD: 225×10^3^ and 230×10^3^±32×10^3^ cells) (**Fig. 3B**). No significant effects on BAL total cell count were observed in TRAIL-treated control healthy mice (median and mean ± SD: 220×10^3^ and 223×10^3^±20×10^3^ cells). Moreover, the nature of the cell populations characterizing BAL samples was analyzed by multiparametric flow cytometry: according to the FSC/SSC gating, lymphocytes (T and B) were identified as CD3^+^ and CD19^+^; granulocytes were identified as CD11c^-^/MHCII^-^; neutrophils were identified as CD11c^-^/MHCII^-^/Gr1^+^/CCR3^+^; eosinophils were identified as CD11c^-^/MHCII^-^/Gr1^dim^/CCR3^+^; alveolar macrophages (AM) were identified as CD11c^+^/MHCII^dim/neg^; dendritic cells (DC) were identified as CD11c^+^/MHCII^+^ (**Fig. 3C**). OVA-mediated airway inflammation was characterized by a significant increase of eosinophils and neutrophils in BAL samples, with respect to the healthy control mice (**Fig. 3D**).

Of note, the prophylactic i.n. treatment with recombinant TRAIL in OVA-challenged mice while did not significantly affect the OVA-induced IgE up-regulation (revised **Fig. 3A**), it was associated to a significant (p<0.01) reduction in BAL cell counts (**Fig. 3B**), with a specific decrease of the eosinophilic and neutrophilic populations compared to OVA-challenged mice (**Fig. 3D**). Consistently, histological analysis showed a reduction in TRAIL treated mice of the morphological features characterizing the OVA-treated mice, such as airway remodeling and inflammation (**Fig. 3E**). Of interest, in our experimental model we found optimal activity of TRAIL in preventing the increase of BAL leukocytes when TRAIL was i.n. instilled 24 hours before OVA challenges, while the protective effect was attenuated when recombinant TRAIL and OVA challenge were administered at the same time (**S1A**–**B Figure**).

### Pretreatment with TRAIL down-modulates CXCL-1/KC in the context of allergen-induced airway inflammation

**Figure 4:**
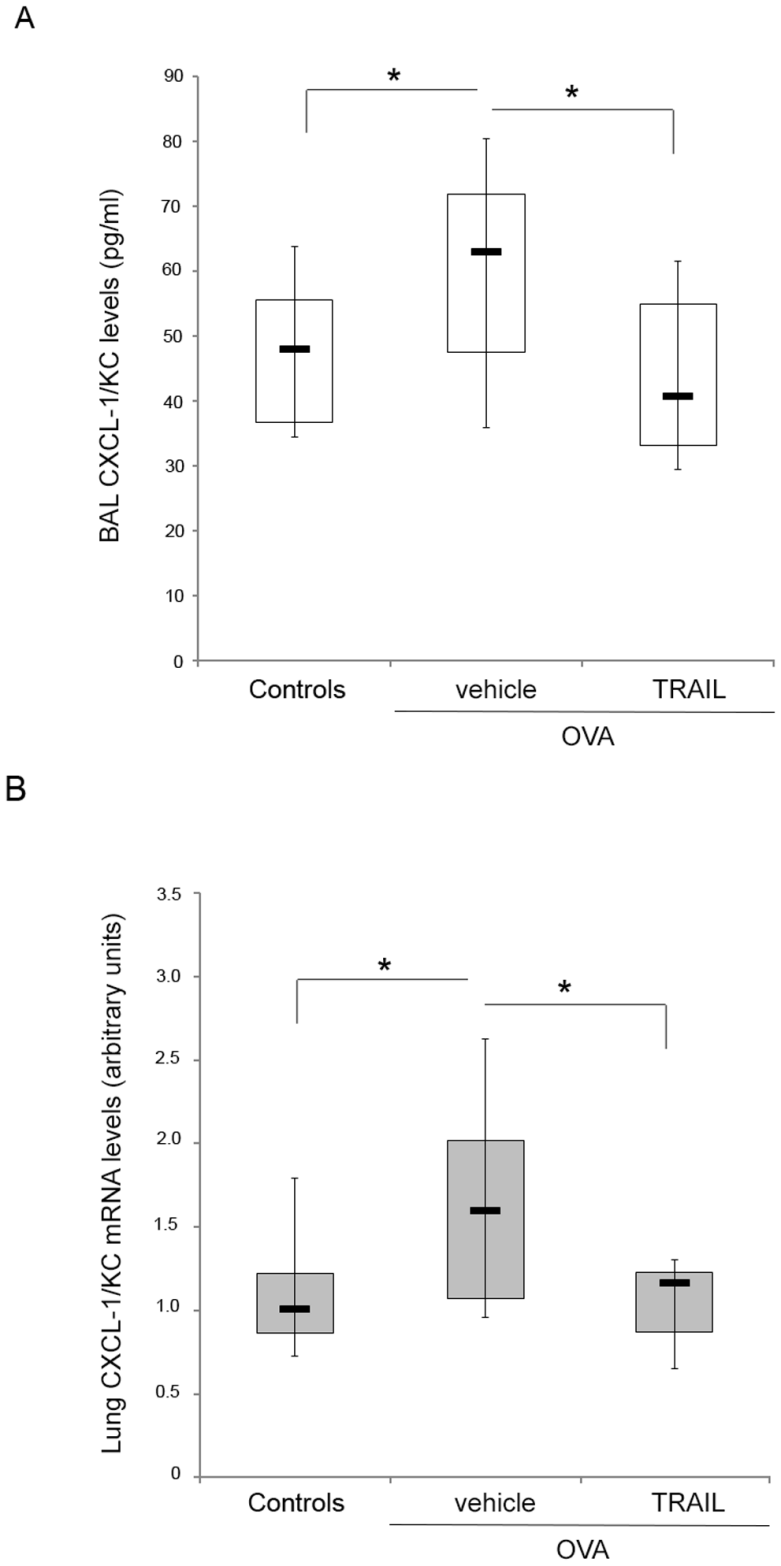
Effect of recombinant TRAIL on OVA-induced CXCL-1/KC expression. In **A**, the levels of CXCL-1/KC in the BAL fluids of Controls, OVA- and TRAIL+OVA-mice were measured by Multiplex assay. In **B**, the expression levels of CXCL-1/KC mRNA in lung tissue of Controls, OVA- and TRAIL+OVA-mice were determined by quantitative RT-PCR. Results from amplifications, done in duplicate, are expressed as arbitrary units after normalization for the housekeeping gene. In **A–B**, horizontal bars are median, upper and lower edges of box are 75th and 25th percentiles, lines extending from box are 10th and 90th percentiles; *, P<0.05 (Mann-Whitney rank-sum test).

Since it has been recently shown that recombinant TRAIL administration might participate to the resolution phase of asthma, by promoting apoptosis of infiltrating leukocytes [9], we next evaluated whether the decreased number of BAL infiltrating cells in OVA-challenged animals i.n. pretreated with TRAIL might be due to induction of apoptosis. Our analyses, although characterized by a high variability from one experiment to the other, did not show significant differences in the percentage of apoptotic cells between OVA- and TRAIL+OVA-mice in our experimental model. Thus, in order to further explore potential mechanisms underlying the effect of TRAIL in this context, we next assessed the BAL fluid samples for a commercial panel of 32 cytokines and chemokines involved in the inflammatory process and that might act as biochemical mediators of the disease (**S1 Table**). Within the cytokines and chemokines detectable in BAL samples at day 32, only the release of CXCL-1 (murine keratinocyte-derived chemokine KC) was significantly (p<0.05) increased in OVA-mice compared to healthy controls (**S1 Table**). Of interest, as shown in **Fig. 4A**, the pretreatment with recombinant TRAIL significantly decreased (p<0.01) the BAL release of CXCL-1/KC (median and mean ± SD: 40.7 and 44.3±14.6 pg/ml) compared to OVA-mice (median and mean ± SD: 63.0 and 60.1±16.9 pg/ml), showing levels comparable to those measured in healthy control mice. These findings were confirmed also at transcriptional level by analyzing the lung levels of CXCL-1/KC mRNA assessed by real time PCR (**Fig. 4B**; controls, median and mean ± SD: 1.01 and 1.1±0.4 arbitrary units; OVA-vehicle, median and mean ± SD: 1.6 and 1.7±0.8 arbitrary units; OVA-TRAIL, median and mean ± SD: 1.2 and 1.1±0.3). In parallel, the analysis of the expression levels of CCL20 and MID-1, proposed as molecular mediators of allergic airways disease [4], [6], confirmed their significant upregulation in OVA-treated mice compared to controls, but did not shown any modulation in TRAIL-OVA mice compared to OVA-treated mice (**S2 Figure**).

## Discussion

Previous studies comparing the TRAIL-/- knock out mice with the wild-type littermates [4], [6], [27] have shown that endogenous TRAIL is involved in the development of experimental asthma. On the other hand, a recent study published while this manuscript was in preparation documented a protective role of exogenous soluble TRAIL in the resolution phase of asthma in a chronic model of allergen inhalation [9]. In this context, the major findings of our current study performed on a model of mild asthma induced by OVA are the following: (i) i.n. pre-treatment with recombinant TRAIL significantly counteracted the increase of infiltrating leukocytes, and in particular of neutrophils and eosinophils, in BAL of OVA-treated mice. Of note, when TRAIL administration occurred simultaneously with OVA challenges, the protective effect of TRAIL was attenuated; (ii) the reduced levels of BAL leukocytes in TRAIL-pretreated allergic animals were not accompanied by increased levels of cell apoptosis. In this respect, our data differ from those of two previous studies which proposed that TRAIL could either promote eosinophil survival [2] or on the contrary eosinophil apoptosis [9]; (iii) among the 32 cytokines/chemokines investigated in BAL fluid, only CXCL-1/KC showed a significant and reproducible increase in OVA-treated allergic mice with respect to healthy control mice and TRAIL pretreatment completely counteracted its increase in both BAL and lung RNA samples. Due to the key role of CXCL-1 in recruiting cellular mediators of inflammation [28]–[34], the ability of TRAIL to counteract the increased production of CXCL-1/KC induced by allergen stimulation likely represents a plausible mechanism accounting for the decreased infiltration of neutrophils and eosinophils in the BAL of OVA-treated animals. Certainly, we cannot exclude that other cytokines/chemokines are involved in mediating the recruitment of neutrophils and eosinophils. However, the specific chemoattractants, and the order in which they act, might differ depending on the specific pathological stimulus and/or the different experimental settings, including the specific biologic assays employed for their detection [27], [31]. Since it is increasingly recognized that both eosinophils and neutrophils play an important role in the asthmatic response in mice [35], the ability of TRAIL pretreatment to down-regulate CXCL-1 and to reduce both eosinophil and neutrophil infiltration is interesting. In this respect, to strengthen the notion that CXCL-1 plays an important role in asthma, a recent study have started the investigation of the potential clinical benefits of a selective CXCR2 receptor antagonist in patients with severe asthma [36].

We believe that our current data might contribute to conciliate the discrepancies between the data of Weckmann et al. [4] and Collison et al. [5], [6], [27], demonstrating that TRAIL-/- knock out mice show less severe inflammatory reaction in asthmatic models with respect to TRAIL+/+ littermates, with those of Faustino et al. [9], suggesting that treatment with recombinant TRAIL exhibits a protective role in allergic airway inflammation. Certainly, these discrepancies could be explained in part by the use of different challenge schedules with OVA in these studies and/or to a different activity between endogenous TRAIL expression and recombinant soluble TRAIL. In this respect, in a completely different setting such as atherosclerosis, while some authors showed increased levels of TRAIL-positive leukocytes in atherosclerotic plaques and proposed a role of TRAIL-expressing leukocytes in mediating apoptosis of vascular smooth cells and plaque destabilization [37], we and other authors have demonstrated a protective role of exogenous TRAIL for the development of atherosclerotic lesions [38]–[40]. Moreover, our current findings underline that the time/schedule of TRAIL administration has a critical role in determining the potential effects of recombinant TRAIL. Indeed, in our experimental model we found optimal activity of TRAIL in preventing the increase of BAL leukocytes (and down-modulation of CXCL-1/KC) when TRAIL was i.n. instilled 24 hours before OVA challenges, while the protective effect was attenuated when recombinant TRAIL was administered at the same time of the OVA challenges. Thus, while there is no doubt that endogenous TRAIL has a permissive role in the induction of experimental asthma [4]–[6], [27], emerging evidences [9], including our results, suggest that recombinant TRAIL administration can have a protective role in allergic airway inflammation.

## Supporting Information

S1 Figure **Effect of recombinant TRAIL on OVA induced airway inflammation when administered at the time of OVA challenges.** Mice were treated with recombinant TRAIL at the time of OVA challenges and the cellular influx in the BAL was assessed. In **A**, the schedule and the timeline of recombinant TRAIL treatments with regard to OVA sensitizations/challenges of BALB/c mice are shown. In **B**, total cell count of cells present in BAL fluids of Controls, OVA- and TRAIL+OVA-mice is shown. Horizontal bars are median, upper and lower edges of box are 75th and 25th percentiles, lines extending from box are 10th and 90th percentiles; *, P<0.05 (Mann-Whitney rank-sum test).
(TIF)

S2 Figure **Expression of CCL20 and MID-1 in the OVA induced airway inflammation model.** The expression levels of CCL20 (**left panel**) and MID-1 (**right panel**) mRNA in lung tissue of Controls, OVA- and TRAIL+OVA-mice were determined by quantitative RT-PCR. Results from amplifications, done in duplicate, are expressed as arbitrary units after normalization for the housekeeping gene. Horizontal bars are median, upper and lower edges of box are 75th and 25th percentiles, lines extending from box are 10th and 90th percentiles; *, P<0.05 (Mann-Whitney rank-sum test).
(TIF)

S1 Table **Levels of cytokines and growth factors analysed by multiplex assay.**
(DOCX)
