# Supplementary figures and images for: Intranasal Administration of Recombinant TRAIL Down-Regulates CXCL-1/KC in an Ovalbumin-Induced Airway Inflammation Murine Model
Source: PLoS One. 2014 Dec 15;9(12):e115387. doi: 10.1371/journal.pone.0115387 (PMC4266651; doi:10.1371/journal.pone.0115387)

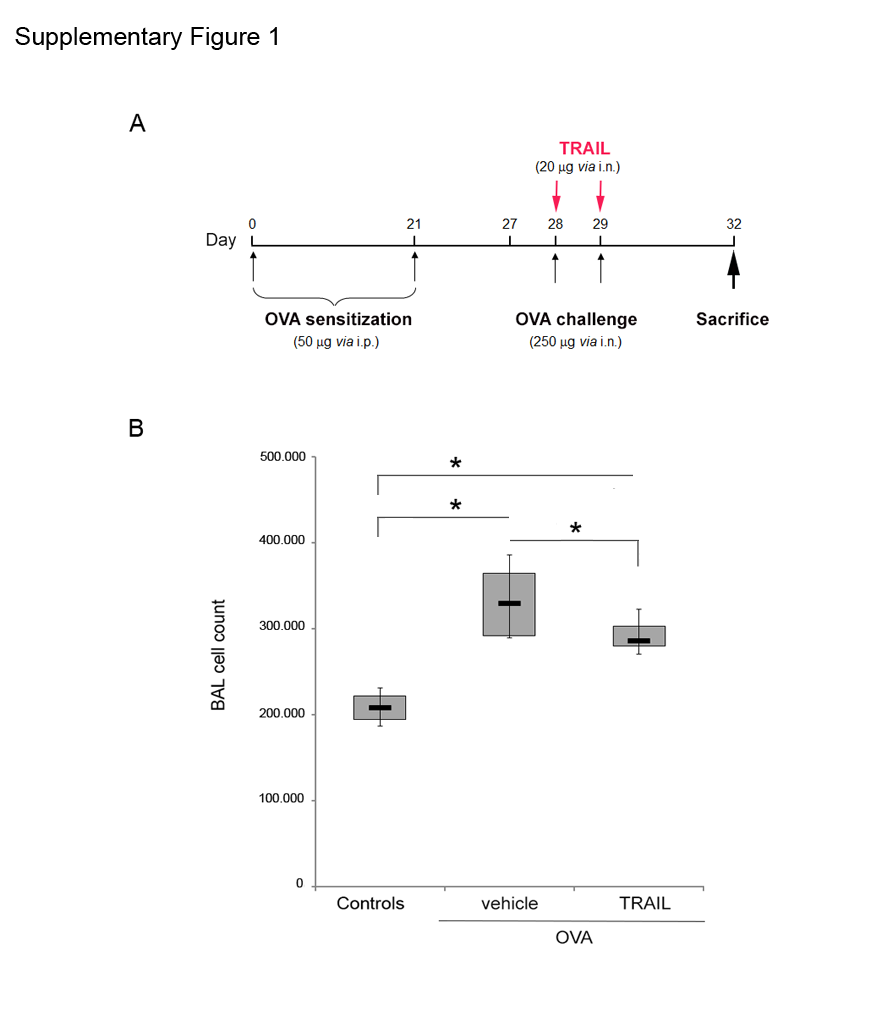

Supplement: S1 Figure — Effect of recombinant TRAIL on OVA induced airway inflammation when administered at the time of OVA challenges. Mice were treated with recombinant TRAIL at the time of OVA challenges and the cellular influx in the BAL was assessed. In A, the schedule and the timeline of recombinant TRAIL treatments with regard to OVA sensitizations/challenges of BALB/c mice are shown. In B, total cell count of cells present in BAL fluids of Controls, OVA- and TRAIL+OVA-mice is shown. Horizontal bars are median, upper and lower edges of box are 75th and 25th percentiles, lines extending from box are 10th and 90th percentiles; *, P<0.05 (Mann-Whitney rank-sum test). (TIF) [file pone.0115387.s001.tif]

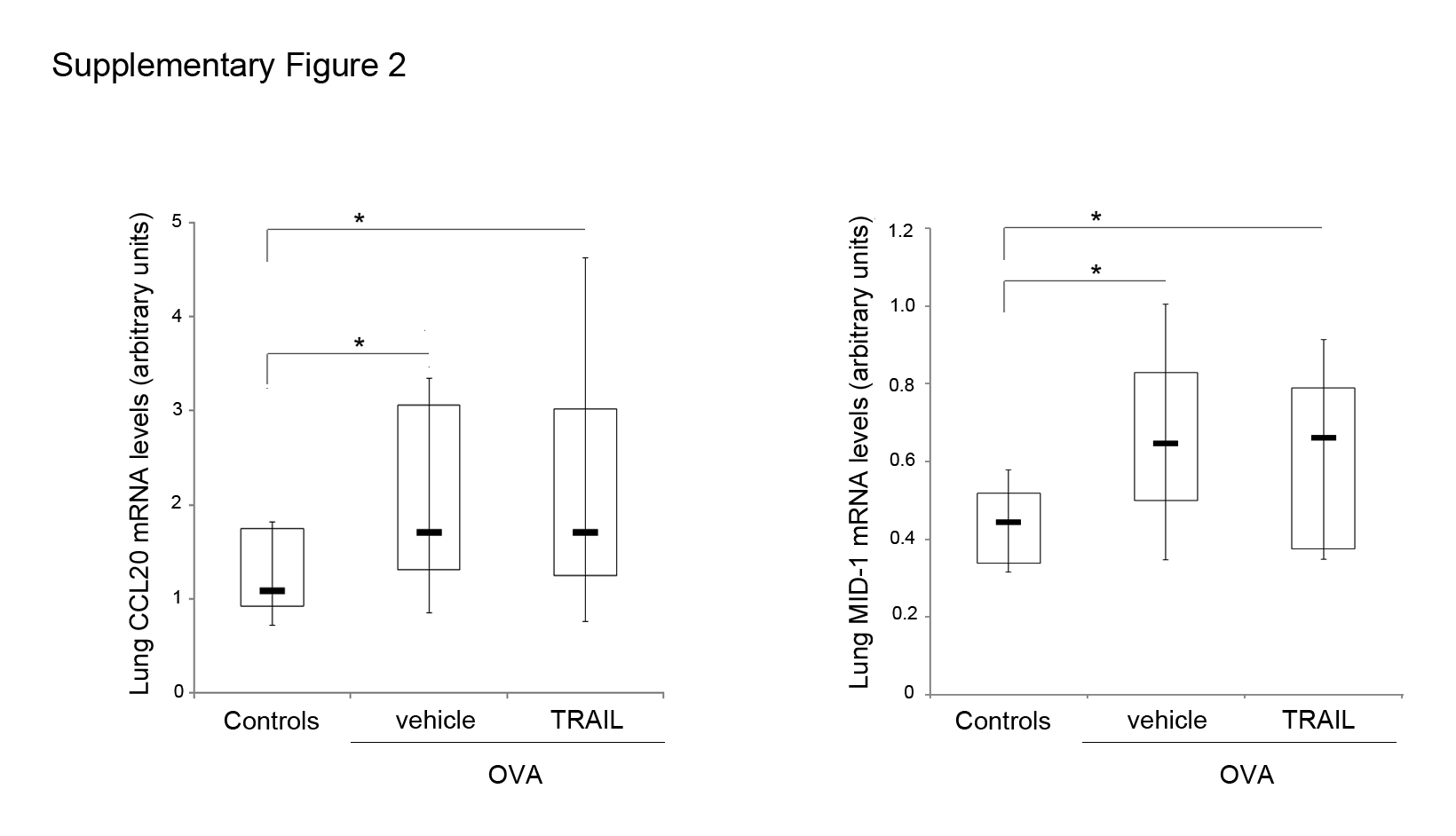

Supplement: S2 Figure — Expression of CCL20 and MID-1 in the OVA induced airway inflammation model. The expression levels of CCL20 (left panel) and MID-1 (right panel) mRNA in lung tissue of Controls, OVA- and TRAIL+OVA-mice were determined by quantitative RT-PCR. Results from amplifications, done in duplicate, are expressed as arbitrary units after normalization for the housekeeping gene. Horizontal bars are median, upper and lower edges of box are 75th and 25th percentiles, lines extending from box are 10th and 90th percentiles; *, P<0.05 (Mann-Whitney rank-sum test). (TIF) [file pone.0115387.s002.tif]
