# Supplementary material for: Intranasal Administration of Recombinant TRAIL Down-Regulates CXCL-1/KC in an Ovalbumin-Induced Airway Inflammation Murine Model
Source: PLoS One. 2014 Dec 15;9(12):e115387. doi: 10.1371/journal.pone.0115387 (PMC4266651; doi:10.1371/journal.pone.0115387)
Supplement: S1 Table — Levels of cytokines and growth factors analysed by multiplex assay. (DOCX) [file pone.0115387.s003.docx]

| **Cytokines and growth factors** | | **BAL levels (pg/ml)^a^** | | |
| --- | --- | --- | --- | --- |
|  |  | **Controls** | **OVA** | ***p*** |
| **G-CSF** | Granulocyte colony-stimulating factor | 3.8(3.9±1.2) | 3.8(3.8±1.2) | *ns* |
| **Eotaxin** | C-C motif chemokine 11; eosinophil chemotactic protein | 1.1(2.1±3.7) | 0.4(1.0±1.6) | *ns* |
| **GM-CSF** | Granulocyte-Macrophage Colony Stimulating Factor | OOR< | OOR< | *-* |
| **IFN-γ** | Interferon, gamma | 0.8(1.7±2.0) | 1.8(1.5±1.5) | *ns* |
| **IL-1α** | Interleukin 1, alpha | 25.1(25.6±26.0) | 22.5(22.3±22.5) | *ns* |
| **IL-1β** | Interleukin 1. beta | 3.7(3.0±2.2) | 3.1(3.1±1.8) | *ns* |
| **IL-2** | Interleukin 2 | 4.2(5.1±3.0) | 4.8(4.6±2.6) | *ns* |
| **IL-3** | Interleukin 3 | 0.0(0.3±0.4) | OOR< | *-* |
| **IL-4** | Interleukin 4 | OOR< | OOR< | *-* |
| **IL-5** | Interleukin 5 | 1.7(2.9±3.2) | 2.0(2.2±2.2) | *ns* |
| **IL-6** | Interleukin 6 | OOR< | OOR< | *-* |
| **IL-7** | Interleukin 7 | 1.3(1.3±0.7) | 1.0(0.8±0.6) | *ns* |
| **IL-9** | Interleukin 9 | 1.8(18.1±13.4) | 1.6(13.7±18.7) | *ns* |
| **IL-10** | Interleukin 10 | 0.3(1.8±3.6) | 0.0(0.0±0.1) | *ns* |
| **IL-12 (p40)** | Interleukin 12 subunit p40 | 1.9(1.5±1.5) | 0.3(0.9±1.2) | *ns* |
| **IL-12 (p70)** | Interleukin 12 subunit p70 | 3.6(2.5±1.9) | 2.4(2.3±1.7) | *ns* |
| **LIF** | Leukemia inhibitory factor | OOR< | OOR< | *-* |
| **IL-13** | Interleukin 13 | OOR< | OOR< | *-* |
| **LIX** | CXCL5 | OOR< | OOR< | *-* |
| **IL-15** | Interleukin 15 | OOR< | OOR< | *-* |
| **IL-17** | Interleukin 17 | OOR< | OOR< | *-* |
| **IP-10** | CXCL10 | 4.2(5.2±3.3) | 4.8(5.8±7.3) | *ns* |
| **KC** | CXCL1; GRO alpha | 48.0(48.0±13.0) | 63.0(60.1±16.9) | *0.01* |
| **MCP-1** | CCL2 | OOR< | OOR< | *-* |
| **MIP-1α** | CCL3 | 6.2(7.3±3.7) | 6.4(6.8±4.8) | *ns* |
| **MIP-1β** | CCL4 | OOR< | OOR< | *-* |
| **M-CSF** | Macrophage colony-stimulating factor | 1.2(1.0±0.6) | 1.0(1.0±0.4) | *ns* |
| **MIP-2** | CXCL2; GRO beta | 1.2(4.5±5.3) | 1.7(3.4±3.7) | *ns* |
| **MIG** | Monkine induced by interferon gamma | 2.9(5.4±8.5) | 2.7(3.2±4.4) | *ns* |
| **RANTES** | CCL5 | 2.3(2.5±1.8) | 2.1(2.1±1.1) | *ns* |
| **VEGF** | Vascular endothelial growth factor | 17.3(16.7±4.5) | 15.0(14.5±3.8) | *ns* |
| **TNF-α** | Tumor necrosis factor, alpha | 1.0(1.1±1.2) | 0.6(0.5±0.4) | *ns* |

**Supplementary Table 1. Levels of cytokines and growth factors analysed by multiplex assay**

^a^Values are expressed as Median (Mean±SD); OOR<: out (below) of detection range

P; Statistical significance (Mann-Whitney rank-sum test)
